# Abstracts and Gleanings

**Published:** 1879-11-20

**Authors:** 


### ABSTRACTS AND GLEANINGS.

**Post Partum Hemorrhage.**—Primarily, both in order and importance, preventive treatment stands. This embraces all that close attention to details connected with the termination of labor. The fundus of the uterus should feel the sustaining hand of the doctor all the time. If the patient is multiparous it would be well to ascertain the complications, if any, of former labors. Authorities say that it is thoroughly good practice to administer a dose of ergot just before the delivery of the placenta. In fact, all little measures should be sedulously and intelligently performed.

When prevention has failed, and conservative measures are set at nought, then active treatment commences. Be it known that one of two conditions is the object sought in all treatment, and those are contraction of the uterus or thrombosis. If contraction can be induced by any means, the treatment ends. Some affirm that it can always be done. But should efforts fail, thrombosis is the next and only resource. To induce contraction we have, viz :

1. Uterine pressure and frictions.
2. Removing clots with hand, and overcoming irregularities of contraction.
3. Removing adherent placenta.
4. Excitement and reflex action; and to produce thrombosis.
5. Injection of styptics.

The efficiency of pressure and frictions over the fundus of the uterus is a well attested fact. Well directed efforts in that direction in post-partum hemorrhage have resulted in arousing tonicity in the relaxed uterus. When it does not yield readily, this may be augmented by the introduction of the hand, if need be, into the uterus, thereby exciting contraction and removing clots, retained portions of placenta, etc. Should there be any irregularity in contraction, the hand acts as distensor to remedy it.

Removing adherent placenta is an operation of extreme delicacy, and even danger where the effort is protracted, for to distinguish it from the uterus itself is difficult. But guided by the cord, the fingers should be gently insinuated between placenta and uterus, and remove that which comes off readily, for no force should be exerted.

Excitement of reflex actions is a frequent and fruitful measure. This, you are aware, is done in many ways. The action of cold; of cold and heat alternately, pouring a stream of cold water from above; flapping lower part of abdomen with web towel; ice, or ice water in uterus or rectum; application of child to the breast; squeezing a peeled lemon in uterus, have all been used with varied success. In recent publications, I find recommended by eminent men several new plans, viz :

Dr. Puglese, in the Lyonese Medical Journal. Complete inversion of body. Patient is taken by shoulders, turned in quadrant of a circle, the center being the pelvis. The head is depressed to the floor. Assistants sustain the head while frictions are made. This, as you can see, puts the pelvic circulation far from the heart and against gravity. A case in point is related, where the patient was pulseless and unconscious. She was kept in position one and one-half hours. She became conscious and spoke before the pulse could be felt. In this form of treatment return to horizontal posture must be gradual.

In the Obstetric Journal, of Ireland and Great Britain, as related by Dr. Griffith, we find these measures : To tightly compress aorta between head and spinal column. Also, by introduction of hand into rectum, and with counter hand on abdomen compress the uterus between the two. This acts by producing contraction of uterus as well as mechanical check upon the bleeding.

Prof. Penrose recommends the use of vinegar for the following reasons, viz :

1. Can be easily obtained.
2. It can be instantly and easily applied without special apparatus.
3. It always cures—at least, never failed in his hands.
4. It is sufficient to arouse the most sluggish uterus, and yet produces no injurious irritation.
5. It is an admirable antiseptic.
6. It acts on the mucous membrane as an astringent. It is applied by carrying a saturated rag into the uterus and squeezing it.

If contraction cannot be induced by one or all of these measures, then the production of thrombosis must follow. This is done by injecting a styptic into the uterine cavity. The possible danger forbids use till all else has failed. When resolved to use, make solution, and with a Davidson's syringe introduce nozzle far into cavity to fundus. Throw solution in continuous stream, not forcibly, taking great care to permit fluid to flow out by side of tube. In this way the desired effect may be induced. It must be remembered, in conclusion, that proper stimulation should accompany all treatment at the discretion of the doctor.—Dr. Stubblefield, in *Nashville Med Journal.*

**Therapeutic Effects of the Subcutaneous Injection of Iron, Condurango, etc.**—M. L. Wyschinski having made a number of experiments in the clinic of Professor Laschkewitsch, of Charkhow, communicated the results to the medical society of that city as follows:

1st. *Subcutaneous Injections of Iron.*—Professor Huguenin uses the citro-ammoniacal pyrophosphate of iron in aqueous solution, with the addition of a little albumen in order to make it less irritating. He uses sufficient quantity of the solution to give three grains at a dose. As a rule there is no trouble, but there has been in two or three cases a small abscess form in the neighborhood of the puncture. The effect of iron introduced into the system in this way is extremely good.

One patient with carcinoma of the stomach was so weak as to be unable to leave the bed. After four injections he could get up and walk a little. Another, very anæmic from hematæmesis due to an ulcer of the stomach, gained three and a half pounds in weight during the time in which six injections were given. In a case of anasarca of cardiac origin, three injections proved sufficient to increase the quantity of the urine, and diminish the œdema. In a hysterical patient who was very anæmic, the hysterical attacks diminished in frequency and force five days after the subcutaneous injections of iron were begun. There was shown in every case a decided improvement in the general condition and strength of the patient.

The preparation used by Huguenin is the best for this purpose. Da Costa experimented with dialysed iron but never obtained so good results. He has always produced pain, swelling and small abscesses.

2d. *Condurango Bark.*—The author gives the results obtained in six cases of carcinoma (two of the æsophagus and four of the stomach). In one case the vomiting and pain disappeared. In another case the result was nil. In the last four cases an evident amelioration was noted. The author thinks that the remedy had a favorable influence over the chronic catarrh of the stomach.

3d. *Antihydropine.*—After reading the paper in the Gazette Medicale de Saint-Petersbourg, by Bogomolow and Unterberger, upon the good effects of blatta orientalis in dropsy, the author determined to try the effect of the subcutaneous injection of antihydropine (an extract of the blatta orientalis), in doses of from one to six grains four to eight times a day. Two of the patients had cirrhosis, four had heart disease, and one Bright's disease.

In only one case were good effects noticed. In this one the urine increased in quantity from 650 to 1500 grains. Wyschinski concludes that it is not proved that the good results of the blatta orientalis seen by Bogomolow and Unterberger were due to the antihydropine.

4th. *Sulphate of Zinc.*—This has been employed in Laschkevitsch's clinic for three or four years. The dose is one-fourth grain in a convenient solution. When the pain is great he gives at the same time one-half grain of codeine. The author has seen the result of this treatment a number of times, but he calls particular attention to the case of a young man, sixteen years of age, who for two years had suffered from anorexia, eructations and vomiting. These symptoms invariably appeared from ten minutes to an hour after each meal; weakness and emaciation were extreme. Examination revealed a slight dilatation of the stomach. Two days after beginning the sulphate of zinc treatment, the vomiting became less violent and rarer. His appetite returned at the end of five or six days, and a short time after he left the hospital.

The remedy is useful too in carcinoma of the stomach by its favorable action on the accompanying catarrh. We should not, however, attribute these effects to its astringent qualities, but rather to the irritation of the pneumogastrics which it produces.—*Revue Medicale Francaise et Etrangere.*

**Ovariotomy, Etc.**—Dr. Yandell, in the Louisville Medical News, writes from London: During the past week I witnessed two ovariotomies, and could have seen blood shed brilliantly each day by famous surgeons had I been of sanguine taste. But I went only to see the ovariotomies, because it is a Kentucky piece of carving, and because a distinguished relative has done it so well, and because I was invited by the greatest and by one of the great ovariotomists to see the useful horror done. The first was performed by Mr. Spencer Wells at a private house. It was a good case—no attachments, no applications—a middle-aged patient in fine condition. The tumor was one large cyst and a knot of small ones, together in weight probably forty pounds. It was done under the carbolic spray, Mr. Thornton and Mr. Dees assisting. As deliberate as a sculptor or a painter, Mr. Wells went at his work. Not two tablespoons of blood were shed, and in less than half an hour the operation was over. Bichloride of methylene was the anesthetic used, and it was given without fear, for neither pulse nor pupil were looked to. For ten years Mr. Wells has used it, and never an accident has occurred. Mr. Wells considers it the safest and best of anesthetics. Dr. B. W. Richardson, its discoverer, says that in a hundred thousand cases where it has been used no evil has resulted. This ovariotomy was Mr. Wells's nine hundred and fifty-fifth case! The youngest ovariotomy he ever did was on a child of eight. He has found an ovarian tumor in an unborn infant. Need I say that this was in a post mortem examination? In all my life I have never seen any thing in surgery so perfect, so beautiful as Mr. Wells' operation. Beautiful is the only word that expresses it, though I confess beautiful is a doubtful term to be coupled with surgery.

Mr. Thornton's operation was on a very old woman, toothless and gray, in hospital. The tumor was larger than in Mr. Wells's case, and was attached to the omentum. This and some slight oozing of blood made the operation more tedious than Mr. Wells's, but Mr. Thornton cuts and tears with the knife and hands of an artist. An interesting feature in this case is that in 1859 a rupture of an ovarian cyst occurred while the patient was violently exerting herself. She was very ill for some days, but soon after recovery the fluid was absorbed and her whole abdomen resumed its natural size. Gradually the malady returned. The tumor showed distinctly where the ruptured cyst had been, a thick fibrous growth as large as one's two hands only remaining of it.

Mr. Thornton uses bichloride of methylene wholly in his surgical operations. It is claimed for it that anesthesia under it is less dangerous, more rapid, and less frequently followed by vomiting, etc., than with chloroform or ether.

Dr. B. W. Richardson is now giving nitrite of amyl internally. He uses it in the same cases he first recommended it in by inhalation. He commences with three-drop doses, gradually increasing until the desired effect is produced. He gives it in alcohol and glycerine—say alcohol, three drams; glycerine, five drams; nitrite of amyl, twenty-four drops. In chloroform-poisoning and in sea-sickness he can not see how it can do any good, though he has no experience with it in these troubles. It is scarcely necessary to tell any reader of the *News* that Dr. Richardson is the discoverer of this useful and potent drug. How lovely it does act in spasmodic affections!

At the *conversazione* of the Royal College of Physicians, Dr. Richardson exhibited his curious new invention, the sphygmophone, or pulse-talker. By it you hear the sounds of the heart and the rush of blood in the arteries. I had before seen and listened to it at his house, and I am inclined to believe it will prove a more practically useful instrument than the sphygmograph. Let me say, in passing, that Dr. Richardson confirms my judgment that Pond's sphygmograph is the best yet invented.

Dr. Thomas Fox, the pupil, co-worker and brother of the late good, famous and beloved Tilbury Fox, it will be pleasant for dermatologists to know, is following in the footsteps of his distinguished brother. Already he is favorably known to the profession by his writings in the *Lancet* and other journals. He is a man of superior mind, fine presence and pleasing manners, and is quite worthy of his brother, of whom all know, and of his father, dead since Tilbury, one of the most successful, respected, and useful general practitioners in England. A strong man was the father; a man who commanded the respect of men and won the admiration of women, as Milner Fothergill says.

Mr. Henry Lee still holds to the belief, and is strengthened in it by prolonged experience, that the moist mercurial vapor bath is the best of all treatments for syphilis. I agree with him that where it does not succeed the fault is with the patient or the giver of the baths. Except for its trouble and expense, I am sure it would be in universal use by the profession. Wednesday I go to the christening of his little daughter, six weeks old, and I wish him many more such events. A christening here is not, as with us generally, a mere matter of prayer-books and water; a celebrative dinner, gladdened by the juice of the grape, following the religious rite. Ah, how the blood of the grape does improve one's appetite and enhance one's affection and augment one's gratitude, and increase one's amiability! And yet I am sure the world would be better without it—I mean the world at large.

**After Delivery.**—Following the birth of the child, tying the cord, and delivery of the child to the nurse, we give attention to the delivery of the after-hirth. As soon as the child is born, if there is any fear of hemorrhage, the hand is placed upon the abdomen compressing and gently kneading the uterus until it contracts firmly—in any case this is done as soon as the cord is cut. When we have made the uterus contract firmly we know by its size whether the after-birth has been expelled into the vagina; if not, pressure upon the abdomen is continued, and slight traction is made upon the cord until the uterus contracts. Now a finger is introduced into the vagina along the cord until the placenta is felt, when an edge of it being brought down the woman is asked to bear down, and with moderate traction on the cord it is brought away.

I have not been troubled with attached placenta, and when I hear physicians talk of introducing a hand into the uterine cavity and peeling them off, I say you don't understand your business, and there is some Munchausen in your composition.

I have been called in consultation in several cases of retained placenta. In one I went over one hundred miles, and found that the physician had pulled the cord off, some two days before; there was a very offensive discharge, and the woman was threatened with puerperal fever. Two fingers were introduced through the os, the uterus being pressed down with a hand upon the abdomen, one edge of the loose placenta was brought down, and with gentle manipulation and an effort on the part of the woman, it was all passed in five minutes. In another case, in this city, the placenta had remained for nearly four days, and the odor was the foulest I ever smelled. It required the introductton of the entire hand into the vagina to bring the disgusting mass away. In both cases the physicians in attendance claimed that "the after-birth had grown fast," but in both it was detached, and had probably been after the first two or three hours. The rule is, unbutton the placenta by bringing one edge through the os, and it will readily be brought away.

Neither have I had the trouble with post-partum hemorrhage that I have heard described, and I attribute in part to good management before labcr, during labor, and after labor. If the physician obeys the maxim—"grasp the uterine globe with the hand as soon as the child is expelled, and see that it contracts firmly"—he will avoid the majority of these unpleasant cases. If the placenta is soon removed, as named above, he will avoid others. The remedy I have relied on, when a remedy was needed, is a tincture of the oil of cinnamon, and I assure you it is much more reliable than ergot.

If there is a sudden and profuse flow of blood, and the woman's life is at once endangered, there is but one treatment. Introduce one hand into the uterine cavity, and hold it there until the uterus contracts firmly, the other hand grasping the uterine globe, compressing and kneading it. The man who fools with ergot, electricity, or astringents, is risking the patient's life. I have had but two such cases, yet they both lived.—*Eclectic Medical Journal.*

**Treatment of Cardiac Dyspnœa.**—Prof. See says, in Concours Medical, that in all cases of continuous cardiac dyspnœa he has found iodide of potassium answer very well, especially where the dyspnœic symptoms were combined with a lesion of the tissue of the heart. It is equally useful in valvular lesions. Even if the diagnostic error of mistaking a simple cardiac dyspnœa for true asthma should be committed, the use of iodide of potassium would not be followed by any evil results, as it is an exceedingly useful drug in asthma. The direct effect of iodide in such cases, is the promotion, or rather liquefaction of the bronchial secretion. This greatly facilitates respiration. The dose given by M. See is 1.25 grammes per day; this is gradually increased from 2 to 3 grammes, and is made as follows:

| | | |
|---|---|---|
| ℞ | Iodide of potassium................................ | 10 grammes. |
| | Syrup cort. aurant........................................ | 200 " |

Dose, two to four tablespoonfuls per day. Each spoonful must be dissolved in a tumbler of water.

Patients suffering from heart disease take iodide of potassium very well—better than other patients. The following are the drawbacks of this drug:

1. Bleeding from the buccal membrane, or bronchitis and hæmoptysis in tuberculous patients. (Phthisis is, therefore, a counter-indication for the use of iodide of potassium).

2. Loss of flesh; in fat individuals this is to be regarded as a favorable symptom.

3. Loss of strength; in such cases the treatment must be suspended at once.

4. Loss of appetite.

Opium may be added to iodine, in order to prevent the evil effects of iodine:

| | | |
|---|---|---|
| ℞ | Iodide of potass.................................. | 10 grammes. |
| | Syr. cort. aurant.................................. | 200 " |
| | Ext thebaic.......................................... | 0.10 to 0.15 grammes. |

Dose, from two to four spoonfuls per day. For the ext. thebaic., the syrup papaveris may be substituted (50 grammes).

Opium is given here with a view of making the iodine more easily tolerated, and of diminishing the cough, which greatly inconveniences the patient.

Another very useful combination is that of digitalis with iodine, as the one has a soothing influence on the dyspnœa by acting on the lungs, and the other increases the action of the heart and modifies the arterial tension. The following formula will be found to answer well:

| | | |
|---|---|---|
| ℞ Julep gommeux | 100 | grammes. |
| Iod. of potass | 1 | " |
| Tinct. digit | 40 | gr. |

Or the following formula:

| | | |
|---|---|---|
| ℞ Ext. gent | 0.10 | grammes. |
| Pulv. fol. dig | 0.15 | " |

To take one pill three times daily, together with the sol. of iodine, which we have mentioned above. In cases where patients cannot take digitalis, chloral will be found to be a good substitute. Thus:

| | | |
|---|---|---|
| ℞ Julep gommeux | 120 | grammes. |
| Iod. of potass | 2 | " |
| Chloral-hydrate | 4 | " |

To be taken every two hours during the day.—*London Med. Record.*

**Uterine and Vaginal Applications.**—Dr. Suesserott, Chambersburg, Pa., writes:

"Long familiar with the almost magical effect of a thick cream of subnit. of bismuth, mixed in pure glycerine, when applied to blistered surfaces, burns, and ulcers externally, as well as the soothing and curative action of the salts of bismuth in ulceration of the stomach, I conceived the idea of using it in ulceration of the cervix uteri. A sufficient experience in that direction has convinced me that the result is no less wonderful than when externally applied. This may possibly be no new suggestion to some of your readers. But, acting on the principle of 'proving all things, and holding on to that which is good,' I would earnestly urge upon all gynecologists, who are not fully satisfied with what they are now using, to give it a trial, and report. I have used a thick glycerole of tannin, dry tannin, glycerole of aloes, and the various astringent, sedative and escharotic substances that have, from time to time, been suggested; but there is no one thing that I have applied that gives as much immediate relief as the subnit. of bismuth and glycerine. The congestion of the cervix is at once abated by the glycerine through the exosmotic action that is set up, and the ulcers disappear, as though waved away by a fairy's wand.

"If this article is not already too long you may append my method of making vaginal applications, which I will endeavor to illustrate. I have a glass tube, a little longer than, and sufficiently small to pass into, an ordinary reflecting glass speculum. I take a pledget of absorbing cotton, on to which I have tightly looped a cord of sufficieut length to project beyond the vulva,, when the cotton is in contact with the womb. Having passed the cord through the tube, I draw the cotton in to the extent of an inch or two, so as to have sufficient space for whatever quantity of bismuth and glycerine, tannin, aloes, or whatever I am about to introduce within the vagina. The speculum having been properly applied, so as to bring the ulcerated surface within view, I next insert the tube, with its contents, within the speculum, and with a wire-probe, armed with a disc of metal about three-fourths the diameter of the tube, I press the cotton to its place, withdrawing the tube and holding the application *in situ* until the speculum is partially removed. As soon as the walls of the vagina fall over the cotton, the probe may be taken away and the medicament is just at the spot desired. My custom is to allow it to remain for thirty-six or forty-eight hours, when the cotton is easily removed by means of the cord by the patient herself. After removal I recommend an injection of a saturated solution of borax or slightly diluted whisky, once or twice a day for two days, when I make another application. If the pledget of cotton is too large it will not remain in the place as well as a smaller one; but this must be determined by the judgment of the operator. A glass rod would serve a good purpose in pressing the cotton out of the tube, and where certain articles, such as tinct. of iodine, have been used, would be better than metal. Its liability to fracture in handling is the only objection that could be urged."—*Med. Record.*

**"Tattooing of Nævi."**—At its commencement he passed around the instrument with which he had performed his operation, and which consisted simply of a bundle of coarse needles securely fastened together. It had been constructed by Dr. Sherwell himself. The paper, he stated, was supplementary to one which he had previously read before the New York Dermatological Society, and in which the method had been fully described. He wished now merely to speak of its continued success, and to describe one case in particular where the result had been very satisfactory. The patient was a lady of twenty seven, who, before he took her case in hand, had been so disfigured that it proved not only a source of annoyance, but also of positive detriment to her in obtaining the kind of employment that she was best suited for. The nævus was of the port-wine-stain order, and covered the whole of the chin. There was also a large patch of similar discoloration in the centre of the cheek. The latter was cured after one operation, but four had been performed upon the principal nævus. Two were extended over the whole surface, and two had been confined to those portions where there was the most disfigurement left after the two days' tattooings. In one final operation, shortly to be performed, it was thought that the cure would be completed.

Dr. Sherwell stated that he had not changed his after-treatment since he had written his last paper, and that he was more than ever favorably impressed with the advantages of the application of collodion. As mentioned before, he had hitherto used only an instrument of home manufacture, but at present Tiemann was making one for him which he hoped would prove even more serviceable. He wished it distinctly understood that the only nævi which is claimed to cure in this way were of the cutaneous variety.

In the discussion which followed the paper, Dr. Hardaway spoke in the highest terms of the method of treatment by means of electrolysis, where a number of very fine needles were employed.—*Dr. Sherwell in Dermatological Association.*

**Skin Irritation by the Administration of Drugs.**—Dr. Robert Farquharson, in the British Medical Journal, presents an interesting summary of the effects on the skin of the administration of certain drugs. Arsenic in medicinal doses has been observed to produce herpes, and in larger doses an eczematous eruption. Phosphorus sometimes produces purpura. Iodide of potassium sometimes produces papules, which quickly become pustular. These may develop into ecthyma or bullæ. Bromide of potassium more frequently produces an acne. Nitrate of silver produces an indelible, dull, leaden color. Mercury brings out an eczematous eruption of the skin. Chloral hydrate causes an erythema, scarlet-fever-like eruption, hemorrhagic purpura. Aconite is occasionally attended by an irritable vesicular eruption. Copaiba often produces a sort of urtica. Quinine occasionally causes two kinds of eruptions: The first are erythematous in character, attended by most distressing itching and tingling, resembling scarlet fever both in the appearance of the rash and the free desquamation which follows; the second assumes a more measly aspect, being occasionally papular, but more generally suggestive of urticaria that occurs in discrete rose-red patches, spreading universally over the skin, and occasionally attended with marked gastric disturbance. Strychnia has produced a rash resembling that from quinine. Belladonna may produce a bright red rash, very like scarlet fever. Cod liver oil occasionally causes acne. Salicylic acid has produced a peculiar, bright punctate rash with erythematous patches, eventually surmounted by vesicles, with sore throat and constitutional disturbance resembling scarlet fever.—*N. Y. Ec. Med. Journal.*

**New Treatment of Ozæna.**—For Dr. Massei, ozæna is a parasitic disease, and hence antiparasitic treatment is indicated. He divides his treatment into three stages, according to the following indications: Dilatation of contracted nasal passages, cleansing and disinfection of affected regions, and local medication.

1. *Dilatation.*—When respiration is rendered difficult from contraction of the nasal passages by hypertrophy of the mucous membrane, and by the presence of a considerable quantity of thick and hardened secretion, Massei dilates them by the use of urethral bougies.

2. *Cleansing and Disinfection.*—When the passages are sufficiently dilated, he orders frequent injections through them, by means of Fauvel's retro-pharyngeal syringe, of a very weak solution of salicylic acid (1 part to 500 of water). He considers this solution useful, not only as a disinfectant, but also as an astringent.

3. *Local Alterative Medication.*—After the nasal fossæ are well cleansed, he makes applications, by means of the speculum nasi, of powdered calomel, upon the ulcerated portions of the mucous membrane.

Massei warns practitioners that when the exudation diminishes, there is always a halt in the course of the disease. It is just then that patience must not be lost, but the insufflations rather insisted upon until complete cure is obtained.

Although the present state of science does not permit the full acceptation of the parasitic theory of ozæna, still the excellent results obtained by this Neapolitan practitioner dispose us to follow the treatment which he proposes.—*Annales des Maladies de l'Oreille et du Larynx.*

**An Effective Cathartic.**—The hearty welcome which is extended by all truly scientific medical men to important advances in therapeutics, is our excuse for relating the following: The town of B——, in this State, has two medical men; one a regular graduate, the other a modest "vet.," whose knowledge of cow and horse diseases is briefly summed up in the portentous words, "great experience." Mr. G. was taken suddenly ill. The regular physician was out of town, and not expected to return until the next day. After much solicitation at the hands of the sick man's friends, the "vet." consented to see Mr. G., and prescribe for him. After looking him over carefully, he delivered himself as follows: "Wal! if he was a cow, I should reckon he hed an allfired attack of bilious colic, and I sort of guess I'd give that cow a pint a castor ile and a pound rochelle salts. Seein' as how Mr. G. ain't a cow, I'd a sort a reduce the dose, giving him half a pint a ile and quarter a pound of salts. It's the only thing that'll fetch him." The friends accordingly forced as much of this really valuable prescription into the sufferer as was possible, he being only semi-conscious. The following morning the "vet." called to see his patient, and asked: "Wal! did the medicine have the desired result—did it move him?" "It most certainly did, said a red-eyed friend. "How often?" asked the "vet." Four times—once before death, and threc times afterwards!—*Hospital Gazette.*

**Hot Water in Chancroids.**—I have lately found a new and very valuable therapeutic application of hot water, namely, in the treatment of infecting chancroids, and more especially in that very intractable form—the phagedenic.

My method of procedure is very simple: A piece of sheet lint is made into a pretty solid ball, and being held in a pair of dressing forceps, it is immersed in water not much below the boiling point (in many cases a temperature of 130° or 140°F. will answer), and then this ball of lint is to be pressed forcibly upon the sore. This is repeated daily for several successive days, or until the granulations begin to assume a healthy appearance. As a dressing, simple cerate will suffice, or the sore may be sprinkled with iodoform and covered with dry lint.

The hot water coagulates the albumen in the secretions, and gives to the sore sometimes a whitish appearance, as when nitrate of silver is applied. It is less painful than any of the mineral caustics, and the pain subsides more quickly; and there is no doubt that it destroys the infecting qualities of the sore as thoroughly, while it possesses the great advantage that it does not destroy any of the living tissues.—Dr. Frank H. Hamilton, in *Va. Med. Monthly.*

**Case of Foreign Body in the Stomach.**—I was called, May 28, 1879, to see Willie N., aged four years, who had accidentally swallowed a round flat tin whistle a size larger than a quarter of a dollar. The patient was a very large boy of his age, with rosy cheeks, but at the time of my visit he was pale and quite nervous, and complained of a good deal of pain along the esophagus and in the stomach. Nausea and vomiting had come on, and a direct emetic was given, but no foreign body was ejected. The nausea, however, continued for several days, and he would vomit every time he tried to eat any thing. He took morphia and bismuth, also lime-water and milk. In a few days he was better, and was able to be about all the time. On July 8th he passed the whistle per rectum without any difficulty.

Cases like the above cause a great deal of anxiety, and there is quite often a desire expressed that active operative procedures be instituted, but experience generally proves that it is the best plan to watch and wait, as nature very commonly expels foreign bodies in its own way and in its own time.

I am indebted to Dr. Cummins, of Louisville, for valuable advice in the above case.—*Dr. J. T. Davis, in Louisville Medical News.*

**Gingivitis of Puerperal Women.**—It is known that during pregnancy the gums frequently become red and congested; a slight pressure on them is sufficient to cause a moderate hemorrhage. At a more advanced stage the teeth lose their solidity, become movable, and may be spontaneously shed from the alveolar cavity. Mastication is rendered difficult, but never causes such pain as is common in alveolo-dental periostitis.

In examining the cause of this gingivitis, Dr. Pinard states that Delestre, in his thesis, lays stress on the congestion, tumefaction and softening of the gums during menstruation, which proves that the functional activity of the ovary and uterus may react on the organs of mastication, and predispose them to congestion and inflammation. Previous pregnancies and a bad general condition seem to exert a great influence as predisposing causes.

This affection (puerperal gingivitis) ordinarily appears after the fourth month of pregnancy, and tends to disappear naturally a month or two after parturition. The local treatment consists in touching the diseased parts with a more or less concentrated solution of iodine. with glycerolate of tannin, chlorate of potash, chromic acid, etc. The local treatment which appears more efficacious, however, and is always crowned with success, is the daily application to the healthy and diseased margin of the gums of lint dipped in a solution of chloral and tincture of cochlearia, equal parts.—*N. Y. Medical Record.*

**Nervous Vomiting—Electricity.**—Dr. Semmola, of Italy, gives the results of observations made during several years on the diagnosis and treatment of nervous vomiting. He finds that electricity in the form of the constant current is the most effective remedy. Soon after the first application, the patients can retain food, although for many weeks previously they had always rejected it. He believes, also, that the constant current is not only a sure remedy, but also a valuable means of diagnosis in all cases of chronic vomiting. If the elements of diagnosis be not sufficient to determine whether the vomiting is dependent on some morbid process in the stomach itself, or on reflex action (as from worms or chronic uterine disease), the application of the current at once settles the question. In cases where the vomiting is not exclusively and primarily nervous, its application fails in enabling the stomach to tolerate food.—*Monthly Ab. Med. Science.*

**The Tattooing of Cutaneous Nævi.**—Dr. Sherwell, in the Dermatological Society, described the peculiar method devised by himself for performing the tattooing process, and described the apparatus which he had invented for the operation. The instrument consists simply of half a dozen fine glover's needles, bound together in a bundle with waxed thread, so that their points shall be one to two millimeters apart. This is used either alone or in connection with some irritating substance, as carbolic acid. Many sittings are required, and the operation is a painful one, but the results are satisfactory in all cases of superficial or cutaneous nævi. Of course, when the nævus is of considerable size and deep, and is supplied by large sinuses, the method cannot be practiced. At the conclusion of his paper, Dr. Sherwell brought before the association a female patient, upon whom he had operated by tattooing, for the relief of a disfiguring nævus of the chin. The cure was not quite completed, but thus far the success was undoubted, and the result of the tattooing treatment could not fail to be ultimately successful.—*Medical and Surgical Reporter.*

**Phosphate of Lime.**—This is a medicine much under-valued. It builds up the constitution by aiding digestion and nutrition, and enables the bony system to grow much fatter than without its use. It can be made into a syrup and given to children with rachitis. A fracture of the anatomical neck of the humerus was healed in thirty-two days by its use. Several other fractures were healed in fifteen to twenty-five days, when without it the bony growth would have been much slower. During pregnancy, the lacto-phosphate of lime should be given for the growth of the fœtus, especially in women of such constitutions where the drain on the system is very great, and even then the child will be born sickly and with weak bones.—*Va. Med. Monthly.*

**Syphilitic Neuralgia.**—It should be borne in mind that some cases of obstinate neuralgia are of syphilitic origin. Dr. Higgins recently reported one to the Toledo (Ohio) Medical Association. It was of a man suffering from pain in the right sciatic nerve, recurring periodically every day at the same hour. He treated it with quinine and anodynes for ten days, when he remembered that he had treated the same man some years previously for syphilis, the symptoms being hyperæsthesia of scalp, nocturnal pains and tibial node. The patient had had a sore twenty years before. There was no paralysis. He put him on the iodide of potassium, which relieved him within four days.—*Med. Report.*

**Treatment of Albuminuria by Oxygen.**—Dr. Beaumette calls the attention of the medical profession to the treatment of albuminuria. After unsuccessfully using all other remedies, he made his patient inhale oxygen gas, and in twenty-four hours the albumen disappeared. Other cases were reported in the Bulletin de Therapeutique. After using it, the albumen reappeared in many cases. Dr. Greletty stated that in diabetes it is serviceable—both sugar and albumen diminishing considerably.—*Va. Med. Monthly.*

**Glycerine in Phthisis.**—Dr. Benevente, a specialist in the treatment of phthisis, prefers glycerine to cod liver oil, as the digestion is thereby improved, the diarrhœas are more easily controlled, and the night sweats diminished, as are also the cough and expectoration.—*Va. Med. Monthly.*

